# A Cross-Sectional Study on Whether Comprehensively Gathering Information From Medical Records Is Useful for the Collection of Operational Characteristics

**DOI:** 10.7759/cureus.61641

**Published:** 2024-06-04

**Authors:** Daiki Yokokawa, Takanori Uehara, Yoshiyuki Ohira, Kazutaka Noda, Naofumi Higuchi, Eigo Kikuchi, Kazuaki Enatsu, Masatomi Ikusaka

**Affiliations:** 1 General Medicine, Chiba University Hospital, Chiba, JPN; 2 General Medicine, International University of Health and Welfare Narita Hospital, Chiba, JPN; 3 General Medicine, Kameda Medical Center, Kamogawa, JPN; 4 General Medicine, Kawakita General Hospital, Tokyo, JPN; 5 Pathology/Medical Inspection, Tokyo Metropolitan Tama Medical Center, Fuchu, JPN

**Keywords:** bayes' theorem, clinical support systems, headache, operational characteristics, medical records

## Abstract

This study tests whether comprehensively gathering information from medical records is useful for developing clinical decision support systems using Bayes' theorem. Using a single-center cross-sectional study, we retrospectively extracted medical records of 270 patients aged ≥16 years who visited the emergency room at the Tokyo Metropolitan Tama Medical Center with a chief complaint of experiencing headaches. The medical records of cases were analyzed in this study. We manually extracted diagnoses, unique keywords, and annotated keywords, classifying them as either positive or negative. Cross tables were created, and the proportion of combinations for which the likelihood ratios could be calculated was evaluated. Probability functions for the appearance of new unique keywords were modeled, and theoretical values were calculated. We extracted 623 unique keywords, 26 diagnoses, and 6,904 annotated keywords. Likelihood ratios could be calculated only for 276 combinations (1.70%), of which 24 (0.15%) exhibited significant differences. The power function+constant was the best fit for new unique keywords. The increase in the number of combinations after increasing the number of cases indicated that while it is theoretically possible to comprehensively gather information from medical records in this way, doing so presents difficulties related to human costs. It also does not necessarily solve the fundamental issues with medical informatics or with developing clinical decision support systems. Therefore, we recommend using methods other than comprehensive information gathering with Bayes' theorem as the classifier to develop such systems.

## Introduction

Clinical decision support systems improve clinical decision-making [1] and reduce diagnostic error rates [2]. Further, using natural language processing (NLP) to extract numerous diagnoses and keywords from electronic medical records (MRs) could facilitate the development of clinical decision support systems [3,4]. Prior studies on the creation of disease classification models for various diseases [5-10] used NLP to extract predetermined feature values as keywords.

Developing clinical support systems requires appropriate data and classifiers. Although many machine learning and deep learning models have been published in the last decade, Bayes' theorem remains a classic and powerful clinical tool [11]. This theorem [6,8,12] is often selected as the classifier owing to its similarity to clinicians' reasoning; it requires data in the form of combinations of diagnoses and keywords to calculate operational characteristics, for example, the likelihood ratio (LR). However, the mechanical processing of MRs has some deficiencies. First, non-medical professionals may have difficulty interpreting the terminology in medical data [13]. Additionally, the creation of an expert-level dictionary would be both cost- and labor-intensive [3], and narrative data are not encoded in a format suitable for immediate use, thereby requiring preparation [14]. Furthermore, although humans tacitly understand the context of negative words, the accuracy of machine processing is insufficient to add annotations to extracted keywords, particularly for positive and negative words (P/N assessment) [3], which is critical for clinical support systems.

Under limited data conditions, the first and second problems could be solved by having doctors manually analyze such data; however, with big data, manual analysis is not feasible. Therefore, a more robust data processing methodology is required. Nevertheless, under limited data conditions, another problem arises owing to keyword frequency. Bias in the frequency of each keyword may be problematic. The frequency of character strings in English has a power-law distribution [15,16]. If the frequency of keywords in Japanese MRs has a similar power-law distribution, if the data are long-tailed, there may be a bias regarding each keyword's frequency.

Therefore, even if big data can be collected, this may not increase the number of completed places in the diagnosis and keyword cross table. Therefore, we examined the number and usefulness of operational characteristics obtained from MR data using a cross table created from annotated keywords (AKs) and diagnoses extracted manually from MRs. We also measured the level of equality in keyword frequencies and modeled the probability functions for the appearance of new keywords. Thus, this study aimed to determine whether the comprehensive collection of MRs could contribute to the development of clinical support systems using Bayes' theorem.

## Materials and methods

Setting and participants

This was a single-center study. We retrospectively extracted the MRs of patients aged ≥16 years whose chief complaint was experiencing headaches and who visited the emergency room (ER) at the Tokyo Metropolitan Tama Medical Center between May 1 and June 30, 2014. Approximately 150 patients experiencing headaches visit the hospital every month. The number of patients' records that could be annotated by reviewing all MRs was approximately 300. Patients were included in the study if they visited the hospital on their own or by ambulance. In Japan, pediatricians treat patients under 16 years of age. The hospital under study does not have a pediatric department; therefore, patients under 16 years of age were excluded. Similarly, if patients were in a severe or critical condition, having experienced a stroke or shock based on vital signs and symptoms noted in the ER or ambulance, they were transferred to a critical emergency center, which became responsible for tertiary care, and thus were excluded from the study.

Keyword extraction, P/N word assessment, and name of diagnosis coding

**Figure 1:**
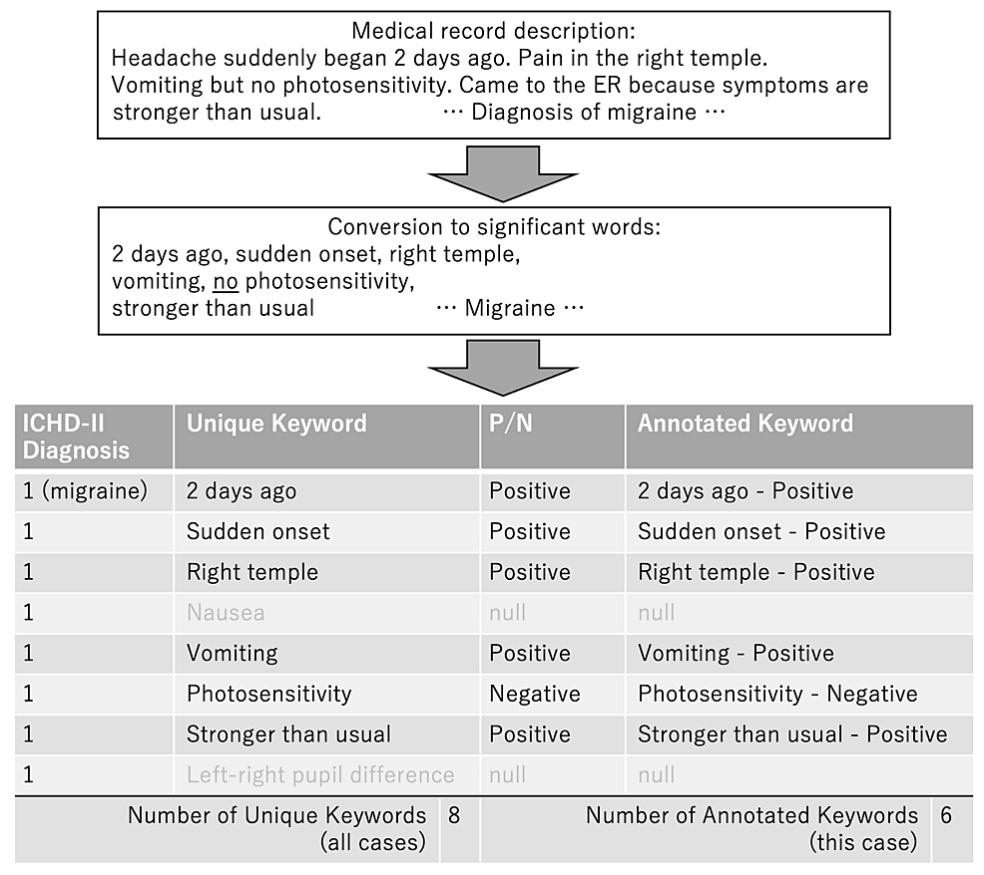
Extraction from natural language ICHD-II: International Classification of Headache Disorders 2nd edition; P: positive; N: negative

Two of the researchers extracted and recorded unique keywords (UKs) from MRs written by doctors (Figure 1). Subsequently, they recorded the results of the assessment of these P/N keywords as AKs. UKs were extracted regarding current and previous medical histories (Hx) and findings of physical and laboratory examinations or imaging (Px). MR entries were not converted, but arbitrary interpretations were eliminated. We generated the AKs via P/N assessment. Referencing previous studies [3,17], assessments were based on the context and presence of negative phrases such as "negative for..." and "…not present." Keywords that did not appear in a case were treated as "no data (null)." AKs were expressed as the total number of keywords that appeared in the text of MRs. The number of UKs and AKs were calculated separately for Hx, Px, positive findings, and negative findings, and the number per case was calculated.

Diagnosis names extracted from the MRs were coded using the International Classification of Headache Disorders 2nd edition (ICHD-II) [18]. Cases in which a diagnosis was not reached, such as "cause unknown," "non-urgent headache," or "non-specific headache," were coded as "Other headaches, head neuralgia, central or primary facial pain (ICHD-II code: 14.0.0)."

Creating the cross table and calculating each UK's operational characteristics

A cross table was created to store the combinations of UKs and ICHD-II diagnoses for all cases. First, true positives (TPs) and true negatives (TNs) were counted using the number of AKs for each ICHD-II diagnosis. Next, TPs and TNs were subtracted from the total AKs, including non-ICHD-II diagnoses, to calculate the number of false positives (FPs) and false negatives (FNs). Operational characteristics (sensitivity, specificity, positive predictive value (PPV), negative predictive value (NPV), positive LRs, and negative LRs) were calculated based on these data. However, because the LRs cannot be calculated unless the TP, TN, FP, and FN values are all ≥1, we determined the number and proportion of combinations for which LRs could be calculated. Furthermore, we examined the operational characteristics that exhibited significant differences in the cross table, using a chi-squared test; subsequently, we calculated their number and proportion.

Creation of keyword histograms and examination of long-tailed distributions

The UKs were sorted in descending order of frequency as AKs to create a histogram. Because the histogram was expected to be long-tailed, the proportion of UKs with a frequency ≥4 or 1 were calculated for both Hx and Px, and the differences in the ratios were examined. Moreover, because UKs can be categorized as positive or negative, these proportions were also calculated and compared between these two groups.

To investigate long-tailed distributions, the proportion of AKs in the top 1%, 10%, and 20% were shown in a histogram ordered by AK frequency, and the differences in their ratios were examined. Additionally, equality of the AKs' frequency was used to examine whether the keyword's frequencies had a long-tailed distribution. Indicators of equality include the Gini coefficient (GC) [19,20]. Larger GC values suggest that the distribution is lopsided and long-tailed [21], whereas smaller values indicate that the distribution is more uniform: 0=perfect equality and 1=perfect inequality. The formula for calculating the GC is as follows: $Gini Coefficient = 2\times \left ( 0.5 - \int_{0}^{1} L(x)dx \right )$. Here, the Lorenz curve is $L(F)=\frac{\int_{0}^{F}x(F')dF'}{\int_{0}^{1}x(F')dF'}$, F(x) is the cumulative distribution function for the keyword frequency, and x(F') is the inverse function of F(x).

In this study, the GC was calculated for the frequency of AKs for each UK. This was calculated separately for Hx and Px and for positive and negative findings.

Regression models of probability functions for the appearance of new keywords

Modeling the increase in dependent variables per case was performed with the frequency of AKs, new UKs, and new ICHD-II diagnoses as the dependent variables and the number of cases as the independent variable. Using linear regression, we calculated the estimated values, standard errors, and coefficients of determination (R_2_) for primary linear, power, and logarithmic functions. Furthermore, considering the model's shape and characteristics, a power function+constant model was created as a form of nonlinear regression, which was used to perform similar calculations. In the nonlinear regression, R2 was calculated as 1 (the residual sum of squares/corrected sum of squares). We entered 10^1^-10^5^ cases into the regression formulas to determine how this changed the dependent variable and concentration values.

Analytics

A chi-squared test was conducted for the operational characteristics, and 95% CIs were calculated. The Clopper-Pearson method was used when there were few samples. Z-scores were calculated by testing differences between the ratios of keyword frequencies and other items. All statistical analyses were conducted using IBM SPSS Statistics for Windows, Version 25.0 (Released 2017; IBM Corp., Armonk, New York, United States). The threshold for significance was set at p<0.05.

## Results

The MRs of 270 patients were analyzed. We extracted 36 diagnoses classified into 26 headache diseases based on the ICHD-II. Table 1 shows the ICHD-II diagnoses and the number of cases. The most frequent diagnosis was "other headaches, head neuralgia, and central or primary facial pain" (ICHD-II code: 14.0.0; 73 cases, 27.0%), followed by "migraine" (ICHD-II code: 1; 36 cases, 13.3%), "tension-type headache" (ICHD-II code: 2; 35 cases, 13.0%), and "headache due to systemic viral infection" (ICHD-II code: 9.2.2; 30 cases, 11.1%).

**Table 1:** Diagnosis names and number of cases ICHD-II: International Classification of Headache Disorders 2nd edition; ER: emergency room; SAH: subarachnoid hemorrhage

Category of diagnoses	Diagnoses by ER doctor (36 types)	Number of diagnoses	ICHD-II diagnosis (27 types)	ICHD-II code	Number of ICHD-II diagnosis	Percentage of total (%)
Primary headaches	Migraine	36	Migraine	1	36	13.3
	Tension-type headache	35	Tension-type headache	2	35	13.0
	Primary headache	19	Other primary headaches	4	19	7.03
Secondary headaches	Post-head, post-neck trauma headache	27	Post-head, post-neck trauma headache	5.1	27	10
	Cervical sprain	1	Acute headache due to whiplash injury	5.3	1	0.37
	Traumatic subarachnoid hemorrhage	1	Headache due to post-traumatic intracranial hematoma	5.5	1	0.37
	Acute subdural hematoma	1	Headache due to subdural hematoma	5.5.2	2	0.74
	Chronic subdural hematoma	1				
	Brain infarction	1	Headache due to ischemic stroke (brain infarction)	6.1.1	1	0.37
	Subarachnoid hemorrhage	1	Headache due to SAH	6.2.2	1	0.37
	Aseptic meningitis	1	Headache due to aseptic (noninfectious) meningitis	7.3.2	1	0.37
	Brain tumor	1	Headache due to brain tumor	7.4	1	0.37
	Hypertrophic pachymeningitis	1	Headache due to other nonvascular intracranial diseases	7.9	1	0.37
	Viral meningitis	4	Headache due to lymphocytic meningitis	9.1.2	4	1.48
	Acute pyelonephritis	1	Headache due to systemic bacterial infection	9.2.1	6	2.22
	Simple urinary tract infection	1				
	Group A beta-hemolytic streptococcus infection	3				
	Peritonsillar abscess	1				
	Influenza B	1	Headache due to systemic viral infection	9.2.2	30	11.1
	Viral infection	29				
	Headache accompanying hypertension	6	Hypertension headache	10.3	6	2.22
	Atlantoaxial joint pseudogout	1	Headache due to cervical disease	11.2	1	0.37
	Acute sinusitis	7	Headache due to rhinosinusitis	11.5	7	2.59
	Dental caries	1	Headache due to disorders of the teeth, jaw, or associated tissues	11.6	1	0.37
	Empyesis	1	Headache due to disorders of other cranial bones, neck, eyes, ears, nose, paranasal sinuses, teeth, mouth, or other structural tissues of the face and head	11.8	2	0.74
	Fibromyalgia	1				
	Headache due to mental illness	1	Headache due to somatization disorder	12.1	2	0.74
	Psychosomatic symptom	1				
	Trigeminal neuralgia	1	Trigeminal neuralgia	13.1	1	0.37
	Greater occipital nerve pain	2	Occipital neuralgia	13.8	2	0.74
	Neuralgia	1	Other headaches, head neuralgia, central or primary facial pain	13.9	1	0.37
	Ramsay Hunt syndrome	2	Headache or facial pain due to herpes zoster	13.15	8	2.96
	Herpes zoster	2				
	Postherpetic neuralgia	4				
	Herpes simplex	1				
Other	Non-urgent headache	73	Other headaches, head neuralgia, central or primary facial pain	14	73	27

UKs and frequencies

There were 623 UKs and 6,904 AKs. Table 1 shows UK and AK frequencies for Hx, Px, positive findings, and negative findings. There were 5.1 times more UKs for Hx than for Px (521 vs. 102) and 2.3 times more for positive findings than for negative ones (552 vs. 237). There were 1.2 times more AKs for Hx than for Px (3,789 vs. 3,115) and 1.5 times more for negative findings than for positive ones (4,164 vs. 2,740). AKs' frequency per case was 1.5 times higher for negative findings than for positive ones (15.42 vs. 10.15).

Proportion of calculable operational characteristics and their contents

Table 2 shows UK and AK frequencies and proportions in the cross table for which the operational characteristics could be calculated. A 623×26 cross table was created (16,198 entries). LRs were calculated for 276 combinations (1.70% of the total); of these, 24 (0.15%) exhibited significant differences, according to the chi-squared test. Table 3 details the operational characteristics of these 24 combinations. For migraines, the keywords nausea, vomiting, and photosensitivity exhibited significant differences.

**Table 2:** Frequency of unique keywords, annotated keywords, and calculated combinations N: number of; PPV: positive predictive value; NPV: negative predictive value; LR: likelihood ratio; Hx: history; Px: physical examinations and tests; Pos: positive; Neg: negative; P/N: positive/negative

Keywords and combination metrics	P/N annotation and operational characteristics	Hx		Px		Hx/Px	Total	
Unique keywords, N	Pos	494		58		8.5	552	
	Neg	142		95		1.5	237	
	Pos/Neg	3.5		0.6		-	2.3	
	Total	521		102		5.1	623	
Annotated keywords, N (per case)	Pos	2,566	9.50	174	0.64	14.7	2,740
	Neg	1,223	4.53	2,941	10.89	0.4	4,164
	Pos/Neg	2.1	-	0.06	-	-	1.5
	Total	3,789	14.03	3,115	11.54	1.2	6,904
Number of calculable combinations, N (ratio to total combinations, %)	Sensitivity	236	1.74	79	2.98	-	315
	Specificity	213	1.57	63	2.38	-	276
	PPV	1,077	7.95	97	3.66	-	1,174
	NPV	530	3.84	678	25.6	-	1,198
	LR	213	1.57	63	2.38	-	276
Total combinations: diagnosis×unique keywords		26×521=13,546		26×102=2,652		-	26×623=16,198	

**Table 3:** Operational characteristics with significant differences The keywords shown in the table represent only those from among the 16,198 entries that exhibited significant differences in likelihood ratios †: significant difference; α: 0.05; CI: confidence interval; Hx: history; Px: physical examinations and tests; PPV: positive predictive value; NPV: negative predictive value; PLR: positive likelihood ratio; NLR: negative likelihood ratio; ICHD-II: International Classification of Headache Disorders 2nd edition

Unique keyword		ICHD-II diagnosis	Operational characteristics (95% CI)					
			Sensitivity (%)	Specificity (%)	PPV	NPV	PLR	NLR
Hx	NSAIDs taken	Other headaches, head neuralgia, central or primary facial pain	86 (49-97)	53 (36-70)	0.30 (0.15-0.52)	0.94 (0.73-0.99)	1.8 (1.1-3.0)^†^	0.27 (0.04-1.7)
	Unilateral	Other primary headache	92 (65-99)	41 (31-51)	0.18 (0.10-0.29)	0.97 (0.86-1.0)	1.6 (1.2-2.0)^†^	0.20 (0.03-1.4)
	Unilateral	Headache due to systemic viral infection	20 (3.6-62)	35 (26-45)	0.02 (0.003-0.09)	0.89 (0.75-0.96)	0.31 (0.05-1.8)	2.3 (1.4-3.9)^†^
	Nausea	Migraine	81 (62-92)	50 (42-58)	0.22 (0.15-0.32)	0.94 (0.86-0.97)	1.6 (1.3-2.1)^†^	0.39 (0.17-0.87)^†^
	Vomiting	Migraine	57 (37-74)	66 (56-74)	0.27 (0.16-0.40)	0.87 (0.78-0.93)	1.6 (1.0-2.6)^†^	0.67 (0.41-1.1)
	Sound sensitivity	Migraine	83 (44-97)	79 (52-92)	0.63 (0.31-0.86)	0.92 (0.65-0.99)	3.9 (1.3-11)^†^	0.21 (0.04-1.3)
	Vomiting	Headache due to head/neck trauma	14 (4.0-40)	58 (49-67)	0.04 (0.01-0.14)	0.85 (0.75-0.91)	0.34 (0.09-1.3)	1.5 (1.1-1.9)^†^
	Fever	Headache due to systemic viral infection	92 (67-99)	67 (51-79)	0.48 (0.30-0.67)	0.96 (0.82-0.99)	2.8 (1.7-4.4)^†^	0.12 (0.02-0.77)^†^
	Joint pain	Headache due to systemic viral infection	89 (57-98)	71 (47-87)	0.62 (0.36-0.82)	0.92 (0.67-0.99)	3.0 (1.4-6.5)^†^	0.16 (0.02-1.0)
	Nausea	Headache due to rhinosinusitis	17 (3.0-56)	44 (36-51)	0.01 (0.002-0.06)	0.94 (0.86-0.97)	0.30 (0.05-1.8)	1.9 (1.3-2.8)^†^
	Sputum	Headache due to rhinosinusitis	80 (38-96)	81 (62-92)	0.44 (0.19-0.73)	0.96 (0.78-0.99)	4.2 (1.7-10)^†^	0.25 (0.04-1.4)
	Nasal discharge	Headache due to rhinosinusitis	80 (38-96)	77 (62-87)	0.31 (0.13-0.58)	0.97 (0.84-0.99)	3.5 (1.7-7.1)^†^	0.26 (0.05-1.5)
	Preceded by common cold	Other headaches, head neuralgia, central or primary facial pain	14 (2.6-51)	38 (21-59)	0.07 (0.01-0.32)	0.57 (0.33-0.79)	0.23 (0.04-1.5)	2.2 (1.2-4.2)^†^
	History of hypertension	Headache due to systemic viral infection	33 (9.7-70)	34 (24-45)	0.04 (0.01-0.12)	0.87 (0.71-0.95)	0.50 (0.16-1.6)	2.0 (1.0-3.8)^†^
	History of headache	Hypertension headache	33 (6.1-79)	24 (16-34)	0.02 (0.003-0.08)	0.91 (0.72-0.98)	0.44 (0.09-2.2)	2.8 (1.1-6.7)^†^
Px	Conjunctival icterus	Headache due to lymphocytic meningitis	50 (9.5-91)	99 (93-100)	0.50 (0.10-0.91)	0.99 (0.93-1.0)	42 (3.7-437)^†^	0.51 (0.13-2.0)
	Pharyngeal erythema	Headache due to systemic bacterial infection	60 (23-88)	82 (69-90)	0.23 (0.08-0.50)	0.96 (0.86-0.99)	3.2 (1.3-8.0)^†^	0.49 (0.17-1.4)
	White spots on tonsils	Headache due to systemic bacterial infection	50 (15-85)	93 (79-98)	0.50 (0.15-0.85)	0.93 (0.79-0.98)	7.5 (1.4-39)^†^	0.54 (0.20-1.4)
	Conjunctival hyperemia	Headache or facial pain due to herpes zoster	50 (9.5-91)	92 (79-97)	0.25 (0.05-0.70)	0.97 (0.86-1.0)	6.2 (1.1-36)^†^	0.54 (0.14-2.2)
	Pharyngeal erythema	Other headaches, head neuralgia, central or primary facial pain	7.7 (1.4-33)	72 (58-83)	0.07 (0.01-0.32)	0.73 (0.59-0.84)	0.27 (0.04-1.9)	1.3 (1.0-1.6)^†^
	Light reflex disorder	Headache due to subdural hematoma	50 (9.5-91)	99 (96-100)	0.50 (0.10-0.91)	0.99 (0.96-1.0)	63 (5.6-668)^†^	0.50 (0.13-2.0)
	Facial sensory disturbance	Headache or facial pain due to herpes zoster	50 (15-85)	98 (94-100)	0.50 (0.15-0.85)	0.98 (0.94-1.0)	29 (5.5-161)^†^	0.51 (0.19-1.4)
	Facial palsy	Headache or facial pain due to herpes zoster	40 (12-77)	97 (92-99)	0.33 (0.10-0.70)	0.98 (0.93-0.99)	13 (3.0-54)^†^	0.62 (0.30-1.3)
	Cranial CT disorder	Headache due to lymphocytic meningitis	50 (15-85)	95 (89-98)	0.29 (0.08-0.64)	0.98 (0.93-1.0)	10 (2.9-39)^†^	0.53 (0.20-1.4)

Long-tailed distribution of keyword frequencies

**Figure 2:**
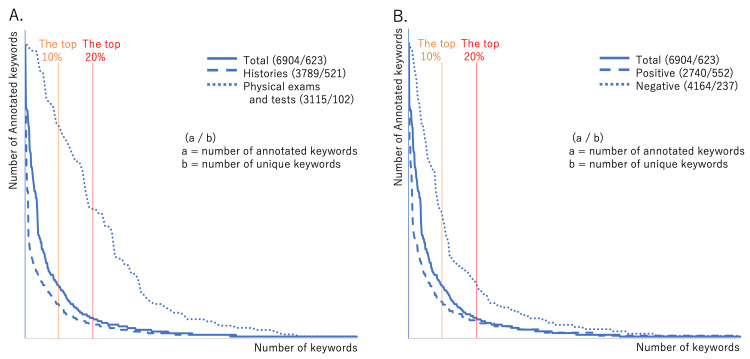
Histograms of annotated keywords (A) Histogram of keywords related to histories and physical exams and tests. (B) Histogram of keywords annotated with positive and negative

**Figure 3:**
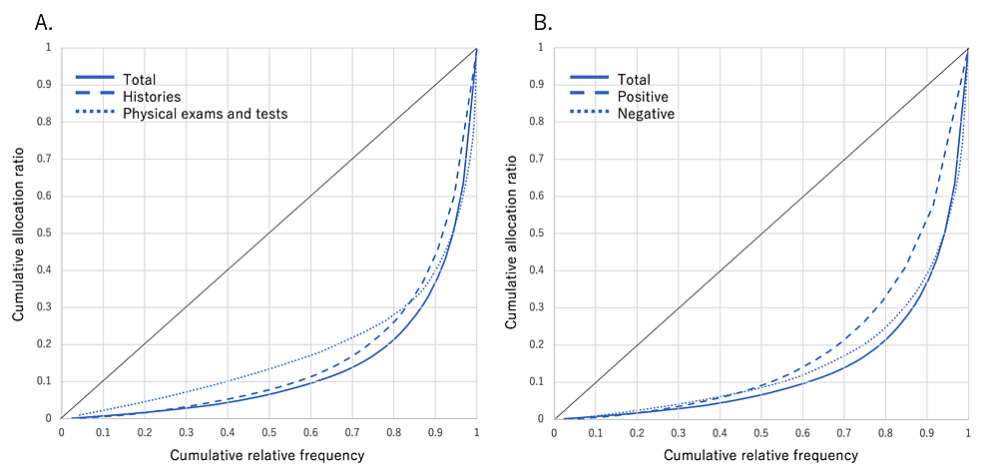
Lorenz curves (A) Lorenz curves related to histories and physical exams and tests. (B) Lorenz curves related to keywords annotated with positive and negative

Figure 2 shows the histograms of each UK's frequency in the MRs (frequency of AKs). The histograms are long-tailed, being more pronounced for Hx and positive findings than for Px and negative findings. Table 4 shows an analysis of the long-tailed distributions of keyword frequencies. The frequencies in the top 20% comprised 78.2% of the total (5,428/6,904). UKs with a frequency of 1 in the tail made up 36.4% of the total. UKs with a frequency ≥4 comprised 43.2% of the total, showing that over half of the UKs' frequency was lower than 4, which is the minimum needed for calculations in the cross table. Hx and positive findings had larger percentages of UKs with a frequency ≥4, compared with Px and negative findings. Table 5 shows the 623 UKs arranged by frequency. The overall GC was high (0.726); a high GC indicates a lack of frequency equality, with large disparities in the number of keyword frequencies. This finding is consistent with long-tailed histograms. Figure 3 shows the Lorenz curves.

**Table 4:** Analysis of long-tailed distributions of keyword frequencies Hx: histories; Px: physical exams and tests; Pos.: positive; Neg.: negative; UK: unique keyword; AK: annotated keyword; Z: Z score; GC: Gini coefficient

Category of keyword		Number of UKs	Count, (%)		Number of AKs	Number of AKs in the top X% of all UKs, (%)			GC
			≥4	=1		1%	10%	20%	
Hx vs. Px	Hx	521	193 (37.0)	208 (39.9)	3,789	570 (15.0)	2,143 (56.6)	2,798 (73.8)	0.680
	Px	102	76 (74.5)	19 (18.6)	3,115	136 (4.37)	1,211 (38.9)	2,040 (65.5)	0.640
	Z (p-value)	-	6.99 (<0.0001)	4.09 (<0.0001)	-	14.57 (<0.0001)	14.63 (<0.0001)	7.54 (<0.0001)	-
Pos. vs. Neg.	Pos	552	175 (31.7)	233 (42.2)	2,740	381 (13.9)	1,412 (51.5)	1,865 (68.1)	0.618
	Neg	237	145 (61.2)	177 (25.3)	4,164	268 (6.44)	2,249 (54.0)	3,071 (73.8)	0.690
	Z (p-value)	-	7.73 (<0.0001)	8.37 (<0.0001)	-	10.40 (<0.0001)	2.02 (0.044)	5.12 (<0.0001)	-
Total		623	269 (43.2)	227 (36.4)	6.904	834 (12.1)	4,206 (60.9)	5,428 (78.2)	0.726

**Table 5:** Ranking of numbers of annotated keywords Pos.: positive; Neg.: negative

Rank	Keyword	Number			%	Cumulative number	%
		Pos.	Neg.	Total			
1	Nausea	94	77	171	2.48	-	-
2	Left-right pupil difference	1	135	136	1.97	-	-
3	Barre sign	2	132	134	1.94	-	-
4	Ocular motility disorder	0	133	133	1.93	-	-
5	Facial palsy	6	127	133	1.93	707	10.2
6	Vomiting	49	78	127	1.84	-	-
7	Light reflex disorder	2	122	124	1.80	-	-
8	Reduced facial sensation	3	119	123	1.78	-	-
9	Abnormal sticking tongue	1	110	111	1.61	-	-
10	Neck stiffness	1	108	109	1.58	1,301	18.8
11	Cranial CT disorder	5	103	108	1.56	-	-
12	Unilateral	63	37	100	1.45	-	-
12	Articulation disorder	3	97	100	1.45	-	-
14	Curtain sign	1	97	98	1.42	-	-
15	Finger-nose test abnormality	2	92	94	1.36	1,801	26.1
16	Reduced hearing	3	89	92	1.33	-	-
17	Brudzinski sign	6	83	89	1.29	-	-
18	History of headache	64	22	86	1.25	-	-
18	Hypertension	55	31	86	1.25	-	-
20	Conjunctival pallor	4	81	85	1.23	2,239	32.4
21	Conjunctival icterus	2	80	82	1.19	-	-
21	Jolt accentuation	14	68	82	1.19	-	-
23	Pronation-supination test abnormality	1	77	78	1.13	-	-
24	Gait disorder	4	64	68	0.98	-	-
25	Diplopia	1	60	61	0.88	2,610	37.9
50	…	-	-	-	-	3,798	55.0
Total	623 items	2,740	4,164	6,904	-	6,904	100.0

Regression models of probability functions for the appearance of new keywords

Table 6 and Table 7 present the modeling and regression results. Linear regression was possible for increases in the AKs. Modeling new UKs and ICHD-II diagnoses per increase in the number of cases showed that, for both linear and nonlinear functions, the best fit was a power function+constant. In both cases, the exponent of the independent variable was ≤1, and the curve was convex at the top.

**Table 6:** Assigning theoretical values to regression models and equations *In nonlinear regression, R^2^ was calculated as 1−(residual sum of squares/corrected sum of squares) Std. error: standard error; ICHD-II: International Classification of Headache Disorders 2nd edition

Dependent variable (y)	Independent variable (x)	-	Regression models							
		Model	Linear		Logarithmic		Power		Power＋constant	
		Equation	$y=ax+b$		$y=a \ln(x)+b$		$\ln(y)=a\ln(x)+b$		$y=ax^{b}+c$	
Annotated keyword	Cases	Parameter, estimate (std. error)	a	26.0 (0.033)	-		a	1.02 (0.003)	-	
			b	−91.5 (5.14)	-		b	23.2 (0.348)	-	
		R^2^	1.000		-		0.997		-	
Unique keyword	Cases	Parameter, estimate (std. error)	a	1.79 (0.23)	a	142 (2.60)	a	0.470 (0.002)	a	43.4 (1.56)
			b	187 (3.67)	b	−226 (12.2)	b	45.3 (0.428)	b	0.475 (0.005)
			-		-		-		c	7.26 (3.91)
		R^2^	0.956		0.918		0.995		0.998*	
ICHD-II diagnosis	Cases	Parameter, estimate (std. error)	a	0.065 (0.002)	a	5.58 (0.066)	a	0.432 (0.006)	a	11.3 (1.18)
			b	10.46 (0.246)	b	−6.44 (0.313)	b	2.46 (0.67)	b	0.213 (0.012)
			-		-		-		c	−11.6 (1.49)
		R^2^	0.865		0.963		0.954		0.986*	

**Table 7:** Best-fit models to theoretical values for variables ICHD-II: International Classification of Headache Disorders 2nd edition

Theoretical value (y)	Best-fitting model	Theoretical value (x)				
		x=10	x=10^2^	x=10^3^	x=10^4^	x=10^5^
Annotated keyword	Linear	169	2,506	25,882	259,639	2,597,209
Unique keyword	Power＋constant	137	394	1,161	3,451	10,288
ICHD-II diagnosis	Power＋constant	7	19	38	69	120

## Discussion

In this study, 623 UKs, 6,904 AKs, and 26 diagnoses were manually extracted from 270 MRs. A cross table with 16,198 combinations of keywords and diagnoses was created, but only 1.70% of the cross tables were completed. We surmised that a comprehensive extraction of MR information would not suffice to gather all the necessary information required to calculate LRs. We also analyzed the distribution of keyword frequencies in MRs. While the top 20% comprised 78% of the frequencies, the histogram had a long tail, with 36.4% of the keywords appearing only once. Because we could not find any previous reports on GC based on free textual descriptions in electronic MRs, no comparisons were possible. However, the GC of 0.726 indicates extremely high inequality, which is consistent with a long-tailed distribution. Therefore, even with a rise in cases, a uniform collection of keywords crucial for calculating operational characteristics cannot be guaranteed. Thus, the comprehensive collection of big data from electronic MRs may not necessarily contribute to developing clinical decision support systems with high diagnostic accuracy. This limitation is notable when using MRs as a data source for developing clinical decision support systems.

Negative data are considered to have the most important contextual characteristics of all clinical information and contribute greatly to classification accuracy [17,22]. In prior studies, mechanical P/N assessments of medical corpora have been performed in English [10,22,23], displaying precision and recall levels of 84% and 73%, respectively, for positive data and 84% and 82%, respectively, for negative data, which indicates that 20% of findings were misinterpreted [9]. In Japanese, the precision and recall levels of P/N assessments were 85.4% and 79.4%, respectively, for positive data, but only 67.6% and 33.3%, respectively, for negative data [7]. A study that extracted negative English words using an algorithm found that slightly less than half of the medical text contained negative information [24]. In the present study, there were 1.5 times more negative than positive data, which is higher than that in previous studies. It is expected for medical text to contain negative findings; however, the extraction of negative findings is often inaccurate, even when using algorithms [3], which is a limitation of recent NLP methods. The negative findings that previous studies' algorithms failed to extract may have been picked up manually by the present study's analysis.

Many of the unique Hx keywords and AKs indicated positive findings; further, for many combinations, PPV could be calculated only with positive findings (Table 1). The reason Hx had more positive findings could be that Hx is dependent on patients' complaints, which means that symptoms they do not mention are unlikely to be recorded. Additionally, patients tend to express their complaints in unique ways, which could make it difficult to identify them with negative words in the natural language. As Table 2 shows, there were some examples of Hx of negative LRs with significant differences, while, in everyday clinical practice, negative Hx commonly contributes to diagnosis.

Meanwhile, Px had many negative findings, and the NPV could be calculated for many combinations. This is likely because physical examinations generally have low sensitivity and are gathered systematically, which generates numerous negative findings. Systematically gathering results is expected to lead to high levels of equality without a long tail (i.e., a short tail). Visually, Px and negative findings are evident in the shorter tails in Figure 2.

One explanation for the more short-tailed appearance of Px and negative findings in Figure 2 is that these categories exhibited clearer sublanguage characteristics, compared with Hx and positive findings, as they had a stronger limitation on the number of words. Past studies indicate that medical texts constitute a sublanguage [25] (i.e., a language used in certain domains by specialized individuals) [26,27] with a limited number of word frequencies and co-occurrence patterns, namely, closure properties, unlike natural language. This is because sublanguages are used to share information among individuals who have similar training and use the same lexicon [28]. Thus, the individuals who wrote the MRs analyzed in our study also comprise a subgroup.

The theoretical values from the regression equations had a power function for the number of UKs and diagnoses and a linear function for the number of AKs. Collecting more cases, UKs, and diagnoses could increase the number of combinations in the cross table. Collecting more AKs would also increase the number of combinations. However, there are two problems associated with increasing the number of combinations. First, AKs had a long-tailed distribution. The keywords appearing in specialist journals extracted from MEDLINE [29] exhibit a long-tailed distribution. If the distribution of AK frequencies in MRs is long-tailed, that in unexamined MRs are also likely to be long-tailed, which means that regardless of how many MRs are collected, the structure of data accumulating in the head and not in the tail will remain identical. Second, a power-law distribution is the best fit for the probability distributions of newly appearing UKs and diagnoses, while their appearance frequencies decrease as the number of diagnoses increases. This suggests that electronic MRs have sublanguage characteristics: closure properties. Therefore, manual annotation would not increase the subsequent efficiency to increase UKs and diagnosis, even if numerous records are gathered [30].

Thus, we cannot conclude that as the concentration rises and the number of combinations that complete the cross table increases, so will the number of combinations with operational characteristics that have significant differences. In fact, only the extremely low 0.15% combination exhibited significant differences in this study.

Limitations

This was a single-center study, which limits the generalizability of the results. Disease occurrence and the reproducibility of physician examinations are limited, potentially introducing selection bias. Further, the entries in the MRs made by non-neurologists working in the ER may have been inaccurate. Although the headache diagnoses were standardized using the ICHD-II, neurologists were not consulted. Additionally, the study period only lasted two months, which meant we were unable to determine the impact of seasonal diseases, which may have biased the diagnoses' distribution. Moreover, the physical examinations' findings may have been inaccurate. These results may have been affected by cultural background and expressions in the Japanese language. Japanese electronic MRs do not describe phenotypes sufficiently and are not intended for reuse in terms of the quality and structuring of the data [4].

## Conclusions

To obtain accurate data, we improved data source selection, extraction, and pre-processing by using narrative MR data and manual annotations. Despite achieving near-perfect accuracy, some combinations could not yield LRs. This study highlights the limitations of using narrative clinical reports for clinical support systems, advocating for a comprehensive approach. Effective design should integrate multiple strategies, including large language models and expert systems, rather than relying solely on algorithms based on Bayes' theorem or LRs.
